# Self-Developed Testing System for Determining the Temperature Behavior of Concrete

**DOI:** 10.3390/ma10040419

**Published:** 2017-04-16

**Authors:** He Zhu, Qingbin Li, Yu Hu

**Affiliations:** Department of Hydraulic engineering, Tsinghua University, Beijing 100084, China; zhuhe14@mails.tsinghua.edu.cn (H.Z.); qingbinli@tsinghua.edu.cn (Q.L.)

**Keywords:** concrete, thermal behavior, mechanical behavior, cracking, temperature stress testing machine (TSTM)

## Abstract

Cracking due to temperature and restraint in mass concrete is an important issue. A temperature stress testing machine (TSTM) is an effective test method to study the mechanism of temperature cracking. A synchronous closed loop federated control TSTM system has been developed by adopting the design concepts of a closed loop federated control, a detachable mold design, a direct measuring deformation method, and a temperature deformation compensation method. The results show that the self-developed system has the comprehensive ability of simulating different restraint degrees, multiple temperature and humidity modes, and closed-loop control of multi-TSTMs during one test period. Additionally, the direct measuring deformation method can obtain a more accurate deformation and restraint degree result with little local damage. The external temperature deformation affecting the concrete specimen can be eliminated by adopting the temperature deformation compensation method with different considerations of steel materials. The concrete quality of different TSTMs can be guaranteed by being vibrated on the vibrating stand synchronously. The detachable mold design and assembled method has greatly overcome the difficulty of eccentric force and deformation.

## 1. Introduction

Cracking due to temperature and restraint in mass concrete, such as concrete dams, highway pavement, and bridge decks, can be easily observed. It is a critical concern to study the mechanism of temperature cracks through the testing method [1,2]. To investigate the performance of concrete after it is casted, some testing programs, such as the so-called ring tests, plate tests, uniaxial restraint, and substrate restraint tests, were conducted [3]. The cracking reasons may be complex and most of the influencing factors on cracking sensitivity can be investigated at the same time by the uniaxial restraint tests [4]. The temperature stress test (TST) method, by adopting the uniaxial restraint test, is thus developed to determine the mechanical and thermal behavior of concrete. The most widely-used TST devices are cracking frame and temperature stress testing machines (TSTM).

### 1.1. Cracking Frame

Both ends of the specimen for cracking frame are held by two cross-heads [5]. The cross-heads are connected by two longitudinal bars, called steel shafts, that provide restraint. The steel shafts are made of special invar steel whose thermal expansion coefficient is extremely low, approximately 1 × 10^−6^/°C, to eliminate the thermal effect on the specimen. Currently, two types of the concrete temperature control methods are provided, primarily. One type uses a fluid-cooled temperature control formwork, which controls the temperature history of concrete by adjusting the temperature of the flowing fluid in the formwork. The other type uses an air environmental chamber, where the concrete specimen is placed into the small environmental chamber, and the temperature of the concrete specimen is affected by adjusting the temperature and flowing of the air in the environmental chamber [5,6]. The concrete deformation varies as the temperature changes. Temperature stress is produced as the concrete deformation is restrained by the cross-head and steel shafts. Deformation of the steel shaft is measured by the strain gauge to calculate longitudinal restraint stress.

Researchers have carried out many tests for concrete cracking with the cracking frame [5,6,7,8,9,10,11]. Under the assumption that concrete is elastic, the degree of restraint provided by the steel shafts on the concrete can be defined by Equation (1) [6].

(1) $$k_{r}=1-\frac{\varepsilon_{r}}{\varepsilon_{f}}=\frac{100}{1+\frac{E_{c}A_{c}}{E_{s}A_{s}}}$$

where $k_{r}$ is the degree of restraint (%); $\varepsilon_{r}$ is the strain under restrained condition; $\varepsilon_{f}$ is the strain of a free specimen ($k_{r}=0$); $E_{c}$ is the concrete elastic modulus (MPa); $A_{c}$ is the concrete cross-sectional area (m^2^); $E_{s}$ is the elastic modulus (MPa) of the steel shaft; and $A_{s}$ is the cross-sectional area (m^2^) of the steel shaft. In reality, creep and damage could affect the concrete so that $E_{c}$ should be modified to measure the restraint degree. As the concrete elastic modulus increases with the development of age, the degree of restraint is not a constant. Thus, the cracking frame can provide a high degree of restraint condition rather than a full restraint condition.

### 1.2. TSTM

**Figure 1:**
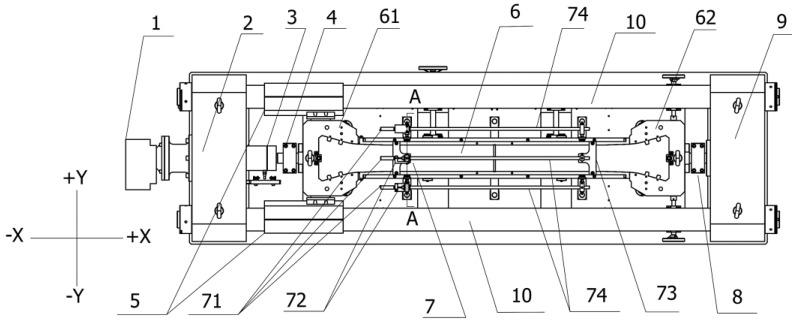
The plan sketch for the TSTM. The numbered parts are as follows: (1) actuating motor, (2) left fixed end, (3) load cell, (4) left snap joint, (5) sliding sleeve, (6) concrete specimen casting region, (61) movable cross-head, (62) fixed cross-head, (7) deformation measuring device, (71) deformation sensor, (72) sensor fixture, (73) embedded rod positioning fixture, (74) deformation transfer guide bar, (8) right snap joint, (9) right fixed end, and (10) steel shaft (developed from the prototype of [12]).

To achieve a certain restraint condition, the TSTM was developed by Gierlinger and Springenschmid [12]. Its schematic drawing is shown in Figure 1. Both ends of the specimen are held by two cross-heads, which are the same as the cracking frame. The concrete specimen casting region is surrounded by the cross-heads and the formworks. The difference with the cracking frame is that two fixed ends are designed and the electrical motor is fixed on one end. The movable cross-head is connected to the electrical motor in order to be adjustable. Two fixed ends are also connected by two longitudinal bars, called the steel shafts, that provide restraint. When a tiny deformation of shrinkage or expansion occurs in the concrete, the movable cross-head will return to the designated position, driven by the motor. The greatest distinction between the cracking frame and the TSTM is the simulation of the restraint degree. The restraint degree of the TSTM is controlled by a computer in a closed-loop manner. The length of the specimen is monitored at a short interval and if the concrete deformation exceeds the control threshold, such as 5 μm for the 1 m specimen, a corresponding controlling order is sent to the motor. Any restraint degree from free deformation to full restraint can be simulated.

### 1.3. The Development of TSTM

The TSTM has been developed by many researchers in recent years and the development history of the TSTM has been reported by Staquet [13]. The designs of TSTMs from different university laboratories differ in some way and the characteristics are summarized below.

1. The deformation measurement method. It is important to determine the value of shrinkage, elastic modulus, and creep. Several methods have been adopted, such as monitoring the displacement of the movable grip [14,15,16,17], however, this method could generate some artifacts by ignoring the interaction between the grip and the sample. Altoubat [18,19] used the linear variable differential transformer (LVDT) attachment method to demonstrate the difference from LVDT on the grip. However, only the displacement of one surface could be measured rather than the whole sample. A method using embedded bars/rods [20,21,22,23] was developed to directly measure the concrete displacement. The embedded bars/rods method has the disadvantage of creating local damage inside the sample, and the strength would be affected. Thus, a deformation measurement method of directly measuring the concrete displacement with little influence on the sample could be developed.
2. Temperature control method. Some devices without thermal regulation systems have been designed to meet the purpose of studying the shrinkage property [18,24,25]. For TSTMs, two types of temperature control methods, the fluid-cooled formwork [26] and environmental chamber [27,28], have been employed. Only a single specimen can be tested in the same environment restrained by the present temperature control method. Concrete is an artificial material having discrete properties, so some parallel specimens should be synchronously performed in the same environmental atmosphere to evaluate the material properties more representatively.
3. Concrete quality. Concrete is poured into the mold of the TSTM directly and a vibrating rod is used to guarantee the concrete quality. When multi-TSTMs are employed, the homogeneity of all samples is hardly to be guaranteed by vibrating the sample separately with a handheld vibration rod. The representation and applicability of the test results will be influenced.

A certain number of concrete samples should be conducted to guarantee that the test results are representative and acceptable. To develop a multi-TSTM system, some designs and methods could be developed.

## 2. The Self-developed Synchronous Closed Loop Federated Control TSTM System

To improve the shortcomings of the TSTM, a synchronous closed loop federated control TSTM system is developed and will be introduced in detail as follows.

### 2.1. The Synchronous Closed Loop Federated Control TSTM System

**Figure 2:**
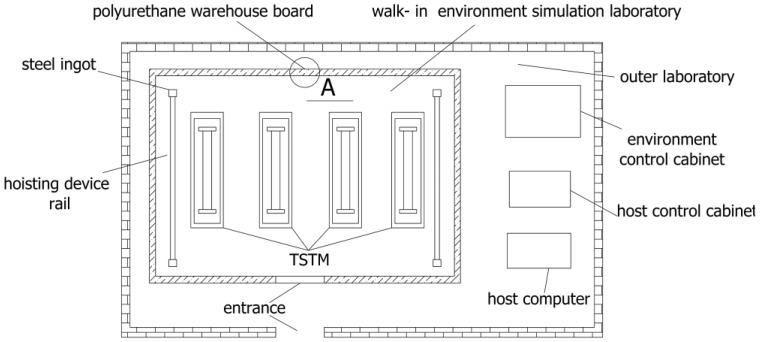
The layout diagram of the laboratory.

**Figure 3:**
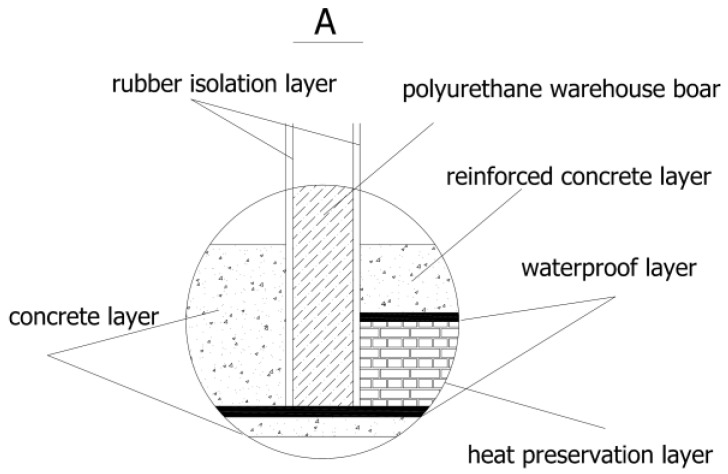
The detailed foundation design.

Referring to Figure 2, a natural environment simulation laboratory system is built, which includes an outer laboratory, a walk-in environment simulation laboratory located in the outer laboratory, a host control cabinet, an environment control cabinet, and a host computer. The environment control cabinet is provided with an environment simulation control unit, which consists of some environmental factor simulation modules, such as temperature, humidity, a carbonization simulation module, spray, and an illumination simulation module. Referring to Figure 3, to prevent the foundation from transferring heat, a heat preservation and waterproof structure is designed. The inner side of the foundation includes a ferroconcrete layer, a waterproof layer, a heat preservation layer, and a concrete layer, from top to bottom. To enhance the heat preservation effect of the laboratory, the wall is configured to be of polyurethane warehouse board. Two rubber isolation layers are pasted to the inside and outside of the wall to shield the vibration from outside of the laboratory.

The walk-in environment simulation laboratory may simultaneously accommodate a plurality of TSTMs therein, and four TSTMs have been placed in the same environmental simulation atmosphere, which can achieve synchronous control of multiple machines. The system is, thus, named the synchronous closed loop federated control TSTM system, and the system has some obvious advantages. First, multi-concrete specimens of temperature stress tests can be conducted synchronously under the same environmental conditions, so that the discretization errors of results can be decreased, enabling the testing results to be more representative and have a broader application scope. The test set parameters of four TSTMs, such as the restraint degree, can be the same or different according to the test requirements. Many parallel tests can be carried out and the test efficiency is improved greatly. Second, no obvious fluctuation of the concrete temperature appears before or after the concrete specimen demolding, which is hardly to be realized by a fluid-cooled formwork [16]. Finally, the steel shaft, steel frame, and concrete specimen of the TSTM are in the same environment. The temperature gradient of the steel shaft and concrete specimen, and even the concrete specimen itself, could be eliminated. However, a new problem is produced where both the concrete and the steel shaft are in the varied environmental atmosphere, and the temperature deformation is inconsistent as the material thermal expansion coefficients of the concrete and the steel shaft are different. The solution will be introduced in Section 2.4 in detail.

### 2.2. The Detachable Mold Design of the TSTM

**Figure 4:**
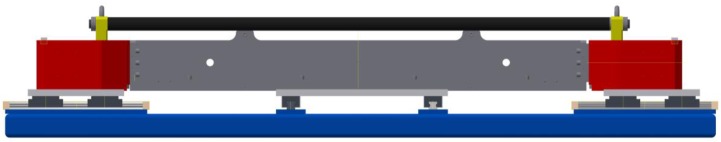
The platform for mold assembly.

**Figure 5:**
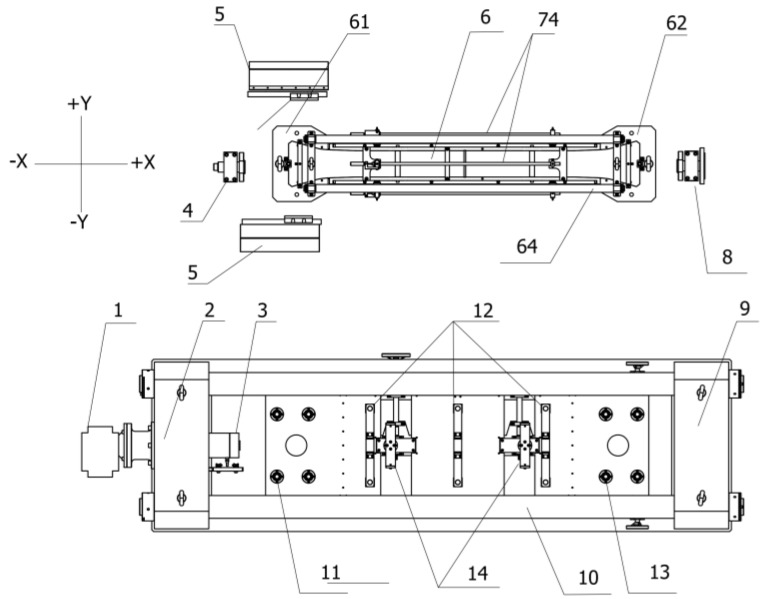
The detachable mold design sketch of the TSTM. The numbers in Figure 5 correspond with those of Figure 1, so the numbers are discontinuous. The numbered parts are as follows: (1) actuating motor, (2) left fixed end, (3) load cell, (4) left snap joint, (5) sliding sleeve, (6) concrete specimen casting region, (61) movable cross-head, (62) fixed cross-head, (74) deformation transfer guide bar, (8) right snap joint, (9) right fixed end, (10) steel shaft, (11) ball supporting points, (12) roller, (13) ball supporting points, and (14) bottom formwork support.

The concrete specimen casting mold is designed to be detachable from the TSTM host part to modify the problems in Section 1.3. Multi-concrete specimens, together with molds, can be vibrated on the vibrating stand simultaneously. However, the centricity may be a new problem caused by the detachable mold design. A mold assembly platform was designed as shown in Figure 4, on which the cross-head can move along the longitudinal direction and the height of the horizontal direction can be kept constant. The cross-head positioning fixture is employed to fix the position of cross-heads. The formworks are then assembled between two cross-heads with bolts. Two supporting bars are located between two side formworks to guarantee the formwork is non-deformable when the concrete specimen is being vibrated. After the concrete specimen is vibrated, the mold will be transferred and assembled on the TSTM host part by the hoisting device. Figure 5 shows the design diagram before the mold is assembled.

The fixed ends are placed on the working platform. Eight height-adjustable ball supporting points and two bottom formwork supports are designed on the platforms, which can allow the concrete to move on it. The movable cross-head and the steel shaft are connected by the sliding sleeve of which only the longitudinal direction (X direction) is allowed. 

The detachable mold design has some advantages. First, multi-concrete specimens can be vibrated on the vibrating stand synchronously, so the quality of multi-concrete specimens can be guaranteed. Parallel tests could be conducted so that results are more representative and test efficiency is greatly improved. Second, formworks can be detached during the test, so that the influence of friction is eliminated. Finally, it is more convenient for the tester to adjust some operations during the experiment.

### 2.3. The Direct Measuring Deformation Method

**Figure 6:**
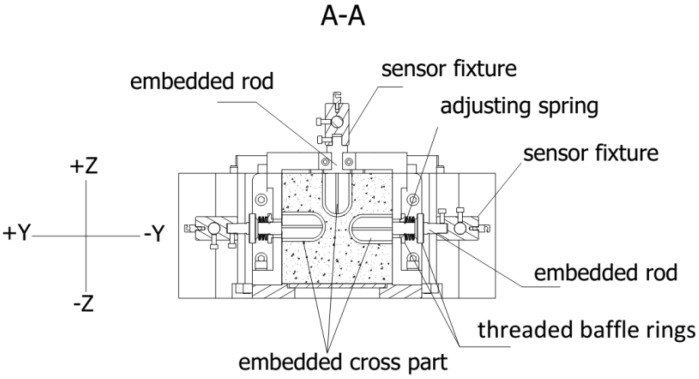
Local detail of the deformation measuring devices.

The design program of the direct measuring deformation method is shown in Figure 1 and Figure 6. Three sets of deformation measuring devices, at the most, could be employed and are located at the top side, left side, and right side. For the top deformation measuring device, the embedded rod is located above the mold cavity and fixed by the embedded rod positioning fixture. The side embedded rod is fixed by a spring adjusting component that comprises two threaded baffle rings and an adjusting spring so that the side embedded rod that can be fixed against the side formwork. It is unnecessary to adopt three sets of deformation measuring devices simultaneously, which depends on the purpose of test. For example, three sets of deformation measuring devices would be adopted to evaluate the deviation of different concrete sides. Only the top deformation measuring devices are allowable for studying the restraint strength and the failure strain because too many embedded parts would cause local damage. The embedded part is designed as a cross shape with steel wire, which has enough stiffness and little volume for reducing the local damage.

### 2.4. The Compensation Method of Temperature Deformation 

All components of the TSTM are in the varied environmental atmosphere, and the temperature deformation is inconsistent as the material thermal expansion coefficients of the concrete and the steel are different. The deformation of the concrete may include the contribution of the temperature deformation caused by the steel shaft and other steel components. A compensation method of temperature deformation is proposed to eliminate the impact of the temperature deformation of the steel components on the concrete specimen deformation. Two different steel materials, 4J36 invar and 45# steel, are adopted. The thermal expansion coefficient is α (1.5 × 10^−6^/°C for 4J36 invar) and α’ (12 × 10^−6^/°C for 45# steel). The steel shaft is made from 4J36 invar and its length is L_1_. The parts of the left fixed end, the right fixed end, and the snap joint are also 4J36 invar and the total longitudinal length is L_2_. The fixed cross-head, the moveable cross-head, and the load cell are made from 45# steel, wherein the total length is L_3_. Referring to Figure 1, if the longitudinal thermal deformation caused by the steel shaft is equal to that of other steel components, the longitudinal thermal deformation caused by the steel of TSTM cannot transfer onto the concrete and its effect could be eliminated. Thus, the concept was proposed that the relationship of the thermal expansion coefficient and the dimensions meets the demand of Equation (2).

α × L_1_ = α × L_2_ + α’ × L_3_ (2)

Finally, the performance parameters of the self-developed TSTM system are listed in Table 1.

## 3. Experimental Program

To verify the ability of the self-developed TSTM system, three series of tests with the same concrete composition were conducted for the purpose of centricity assessment, temperature deformation compensation verification, and temperature stress testing. The raw materials for mixing the concrete are from the Xiangjiaba Dam, which is one of the largest hydropower stations in the world, with a power plant installed capacity of 7.84 million kW. The mixed ratio of the concrete for the experiments in Section 3.1, Section 3.2 and Section 3.3 is listed in Table 2, of which the water-cement ratio is 0.53. The constituents of the Portland cement are listed in Table 3. The fine aggregate (river sand) with a fineness modulus of 2.12 is used and the limestone gravel (with a diameter 5–20 mm) is employed as coarse aggregate. The aggregates are oven-dried at 100 °C for 48 h before the concrete is mixed. The limestone gravel has a dry-bulk density of 2660 kg/m^3^ and a saturated water absorption value of 1.94% by mass, which is determined by the method in [29].

### 3.1. The Centricity Assessment of the TSTM 

**Figure 7:**
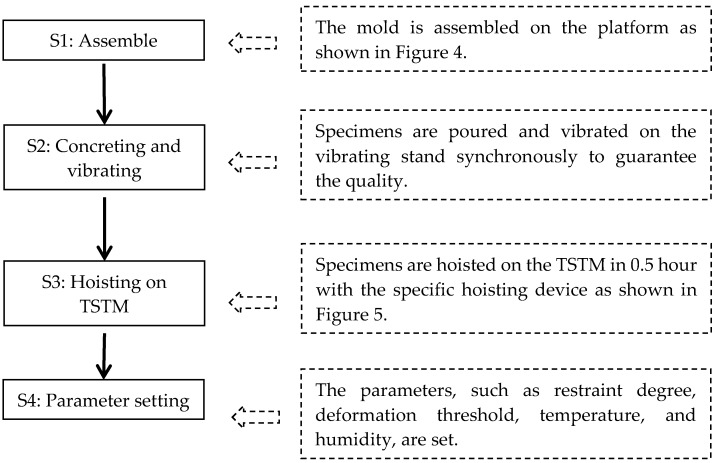
The workflow of the TSTM experiment.

The TSTM has adopted the design of a detachable mold, which has never been reported in traditional TSTMs. The mold and the specimen may be assembled and disassembled at intervals, so the concentricity of the rig is vital to ensure that the load and deformation of the specimen are uniform. Since the designing scheme and the operation processes of all four TSTMs are identical, only one TSTM is conducted for assessing the centricity of loading. Three sets of deformation measuring devices are employed, as shown in Figure 6, and the specific implementation process of the TSTM experiment is shown in Figure 7. The sample is set as a free specimen until the concrete age reaches seven days, and the load is applied to the sample and varied between –35 kN and 15 kN. The 15 kN load represents that the concrete stress is in tension. The beginning load is –25 kN to reduce the gap effect and the strain is recorded as 0 με initially.

A criterion called the eccentricity degree is defined by Equation (3) to evaluate the concentricity of the TSTM.

Eccentricity Degree =│d_top_−d_ave_│/d_ave_ (3)

where d_top_ is the displacement value of the top side, and d_ave_ is the average displacements of the top, left, and right sides. 

### 3.2. Temperature Deformation Compensation Verification Experiment

**Figure 8:**
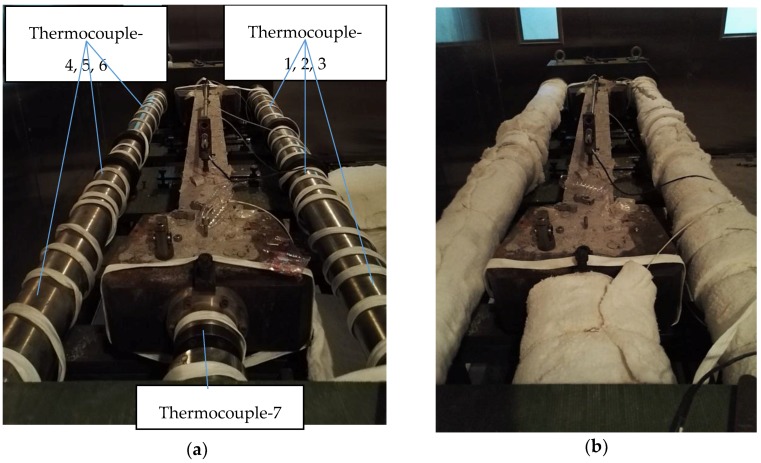
The verification test of the compensation method for temperature deformation. (**a**) A steel shaft wrapped with the electric heating cable. (**b**) The TSTM coated with the thermal insulating material.

To evaluate the effect of the temperature deformation compensation method, the temperature deformation test is designed. The parts of the steel shaft, cross-head, and the snap joint in Figure 1 are wrapped with the electric heating cable, as shown in Figure 8a, and then it is coated with the thermal insulating material, as shown in Figure 8b. Seven thermocouples are attached to the surface of the steel to monitor the temperature of the steel. The age of the concrete sample adopted in the test is three months so that the temperature field of the concrete is stable during the test. The environment temperature is maintained at 20 °C to keep the concrete temperature constant. The steel shaft, cross-head, and the snap joint are heated by the electric heating cable from 20 °C to 70 °C to generate temperature deformation and the heating process is shown in Figure 13. Thus, the concrete deformation caused by the temperature deformation of the steel part can be measured.

### 3.3. TSTM Performance Verification Experiment

The temperature stress test of concrete is conducted and the TSTM performances, such as simulating the different restraint degrees, multiple temperature regulation modes, and closed-loop control of multi-TSTMs, are verified. Four concrete specimens numbered as TSTM-A, TSTM-B, TSTM-C, and TSTM-D are poured into the TSTMs, correspondingly. The thin film is placed between the concrete and the formwork, and the lubricant is used for reducing the friction and making the demolding easier.

- Test Start Time

The method to determine the beginning of the test has been discussed by the researchers. Altoubat [18] states the 12 h is the earliest possible time to apply instruments to the test without damaging the sample. Staquet [13] chooses the start of test at the end of the setting time and the sample is initially restrained by the stiffness of the frame with the motor being turned off until the stress inside the concrete reaches 0.01 MPa. The concrete strength is not high enough to suffer the weight of the deformation measurement device mentioned in Section 2.3 at the very early age. The displacement of the movable cross-head is measured [14] instead of measuring the concrete directly during the first 25 h to avoid local damage near the embedded part. This method is acceptable at the early age as the rigidity of the concrete is very low. The motor is also turned off until 25 h passes to avoid premature damage and the sample is initially restrained by the stiffness of the frame [21,25].

**Figure 9:**
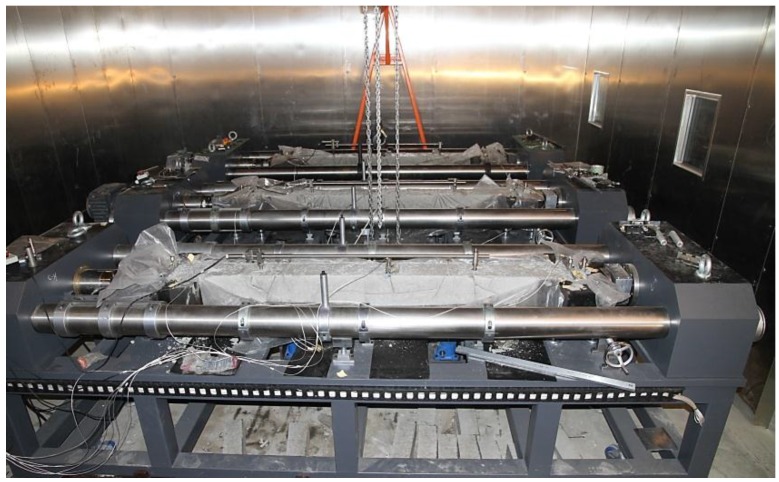
TSTM after the demolding.

After 25 h, the formworks are demolded as the concrete strength has increased and the direct measuring deformation method is adopted by the upper side of the deformation sensor, as described in Section 2.3. The TSTM picture after demolding is shown in Figure 9. As the formworks are removed, the concrete is exposed in the same environment with the machine part of the TSTM. Additionally, the friction is eliminated as the surfaces are no longer restrained by formworks. The measured distance of the specimen is 1000 mm even though the actual length is 2000 mm, obtaining a uniform distribution of restraint stress. The resolution of the deformation sensor is 0.1 μm.

- Multi-TSTM Settings

Four TSTMs are all tested in the same environmental atmosphere, for which the air relative humidity (RH) is 80%. TSTM-A is set as the free specimen and the reference of TSTM-C. The compensation cycle of TSTM-C begins when the increment value of the free specimen strain exceeds 2 με (2 μm for 1 m measured distance) and the restraint degree of TSTM-C is 50%. TSTM-D is set as 100% restrained, which is implemented by the motor adjusting the concrete to its original position when the concrete strain exceeds 2 με.

TSTM-B is also set as an almost free specimen, of which the restraint degree is 5%. To measure the elastic modulus (E), a compressive load about 45 kN (before 90 h) and 60 kN (after 90 h) is applied on the specimen to generate deformation every 12 h, which is called “the active method of elastic modulus determination” [16]. E is calculated by Equation (4):

(4) $$E=\frac{\Delta \sigma }{\Delta \varepsilon }$$

where Δσ is the stress variation and Δε is the stain variation. 

The method called standard method [29] is also employed to determine E. The specimen dimension is 100 mm × 100 mm × 300 mm; and the specimens are all cured in the walk-in environment simulation laboratory which is the same as the TSTM. When the age is 24 h, 48 h, 120 h, and 160 h, three specimens are tested and the average value is adopted as the elasticity modulus of the age. The E of each specimen is calculated by Equation (5):

(5) $$E=\frac{\sigma_{40\%}-\sigma_{0.5MPa}}{\Delta \varepsilon }$$

where $\sigma_{40\%}$ is the stress corresponding to 40% of the failure stress, $\sigma_{0.5MPa}$ = 0.5 MPa, and Δε is the strain variation.

- Temperature Modes

To evaluate the temperature control ability of the TSTM system, three temperature modes are designed. No. 1, from four hours to 70 h, the mode of simulating the semi-adiabatic condition for concrete is designed and the phase is named as the temperature following phase, which is implemented by controlling the environment temperature the same as the internal temperature of the concrete. No. 2, to simulate the temperature history in the dam by water cooling, a designed cooling temperature curve is input and this phase is named as the design cooling phase (70–135 h). No. 3, the cold wave weather case, which is common in dam engineering, is designed by cooling down the environment temperature at the rate of 1 °C/h to obtain a crack (after 135 h).

**Figure 10:**
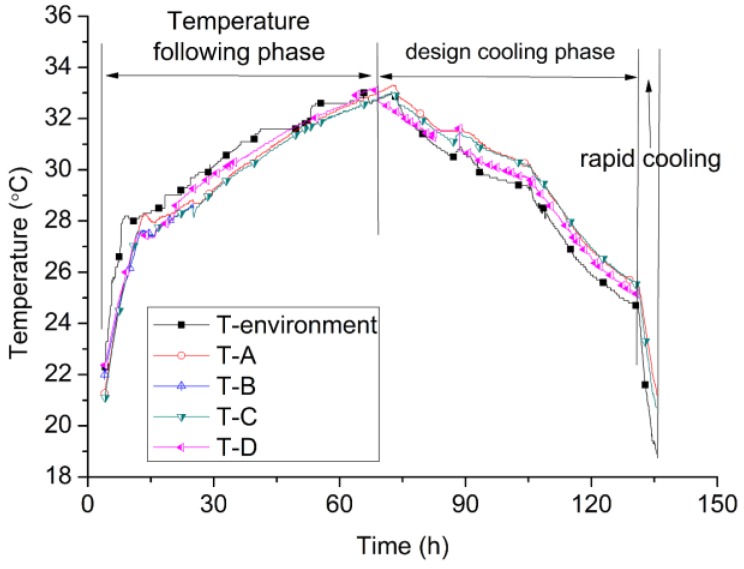
The simulated temperature history curve of the experiment.

The specific processes of the temperature results are shown in Figure 10. Three thermocouples are embedded into the concrete on each TSTM and the average values of each TSTM are named as T-A, T-B, T-C, and T-D, as shown in Figure 10. The T-environment represents the average temperature value measured by four thermocouples located 30 cm above the concrete specimens.

## 4. Results and Discussions

### 4.1. The Deformation Results to Assess Eccentricity Degree of TSTM

**Figure 11:**
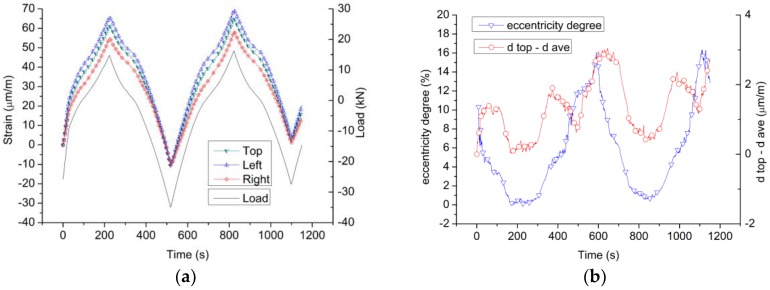
The deformation and eccentricity degree results of TSTM. (**a**) The load and deformation curve of the concrete generated by the actuating motor of TSTM. (**b**) The eccentricity degree of the TSTM.

**Figure 12:**
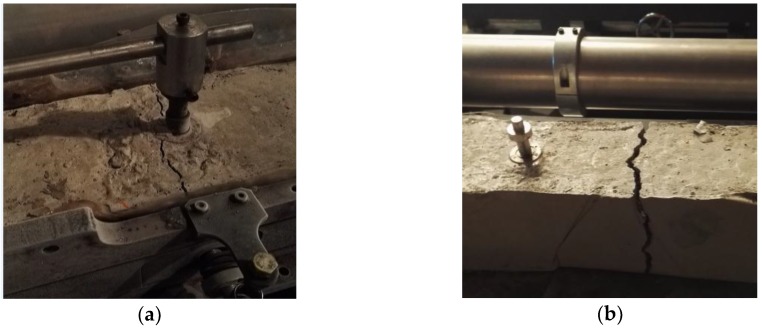
The failure patterns with different deformation measurement methods. (**a**) The sample with top, left, and right embedded parts in Section 3.1. (**b**) The sample with top embedded part in Section 3.3.

The concrete specimen was pushed and pulled by the actuating motor to verify the eccentric degree of the self-developed TSTM. The results in Figure 11a show that the three deformation curves of the sensors located on the top, left, and right sides appear to have the same shape. Additionally, the deformation magnitudes of the three sensors are very close. Figure 11b shows that the maximum eccentricity degree is 16%, and it occurs at the moment that the strain value is low and around 0 με. As we know, the calculation error will be amplified when the denominator magnitude is small. The eccentricity degree is below 10% when the strain is larger than 10 με. Meanwhile, the value of d_top_-d_ave_ ranges between −2 με and 3 με in all loading processes. The difference between the top deformation value and the average deformation value is quite small, which shows that only adopting the top side deformation measuring device during the test is proper to achieve the aim of directly measuring the concrete deformation. After the centricity assessment test, the sample is pulled to failure, and the failure pattern is shown in Figure 12a. Concrete failed at the position near the embedded rods because three sets of embedded parts have affected the integrity of the concrete and caused the local damage. The failure pattern of the sample with only the top embedded parts adopted in Section 3.3 is shown in Figure 12b. The failure position is far from the embedded part and in the middle of the sample where the stress is almost distributed evenly, which indicates that the embedded part designed as a cross shape causes little local damage. The results show that the volume of the embedded part should be designed as small as possible to avoid causing local damage. Only the top deformation measuring device is proposed if the restraint strength and the failure strain are the research points, because three sets of embedded parts will cause local damage and the failure stress will be decreased.

In addition, the shape of the load curve in Figure 11a is also similar to the deformation curves. The deformation shows the synchronicity with the load changes, which indicates that the actuating motor connects tightly with the mold, and the detachable mold design of the TSTM shows good centering performance. With the consideration of a series of design concepts, such as the sliding sleeve on the steel shaft, the height-adjustable ball supporting points, cross-head positioning fixture, and the mold assembly platform, the self-developed TSTM has greatly overcome the difficulty of eccentric force and deformation.

### 4.2. The Results of the Temperature of Deformation Compensation Verification

**Figure 13:**
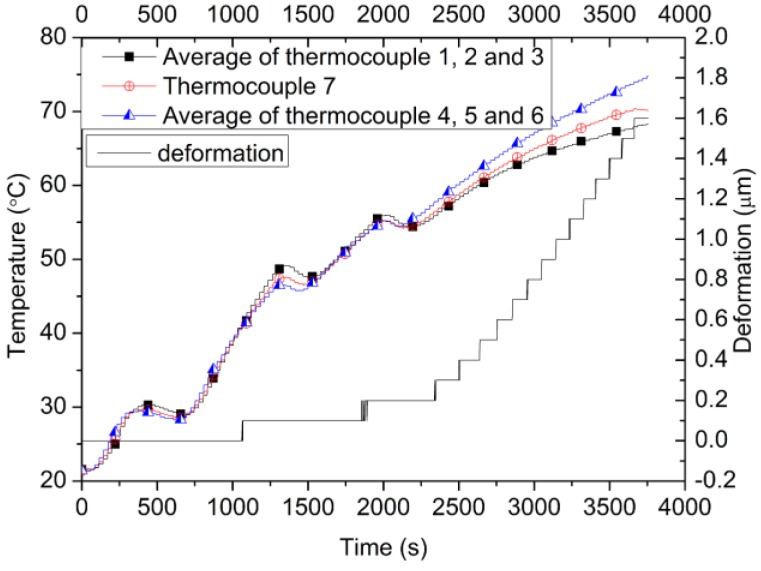
Verification test results of the compensation method for temperature deformation.

As shown in Figure 13, the heating rate is difficult to control quantitatively by adopting an electric heating cable. However, the temperature results of thermocouples 1–7 in Figure 8 are quite similar, especially under 60 °C, which shows that the steel shaft is heated evenly by being coated with the thermal insulating material. The thermal expansion coefficient of Invar is not a constant, but rather a parameter related to the temperature. The average thermal expansion coefficient of the steel shaft adopted by the TSTM system is 1.5 × 10^−6^/°C for the temperature ranging from −20 °C to 100 °C. When the temperature of the steel shaft varies from 20 °C to 60 °C, the concrete deformation varies by only 0.3 μm (the measuring distance is 1 m). However, the temperature deformation of concrete varies from 0.3 μm to 1.6 μm when the temperature increases from 60 °C to 70 °C.

The concrete temperature is kept constant at 20 °C and the testing time is approximately one hour, so that the concrete deformation is only caused by the external temperature deformation. The temperature deformation of the steel shaft is approximately 150 μm as the temperature varies from 20 °C to 70 °C. However, the deformation magnitude of the concrete specimen is 1.6 μm, which is only approximately 1% of the steel shaft, and negligibly small. 

The temperature deformation of the TSTM, mainly contributed by the steel shaft, will transfer into the concrete if the compensation method was not adopted. Consequently, the motor will start, caused by the pseudo-deformation. The results indicate that the compensation method of the temperature deformation with different steel materials combined with each other can eliminate the vast majority of the effects of the external temperature deformation on the concrete specimen.

### 4.3. The Results of the TSTM Performance Verification Experiment

- Temperature Regulation Results

During the temperature following phase, the concrete temperatures of four TSTMs develop synchronization in Figure 10, which shows that the hydration degree and quality of the concrete of the four TSTMs differ slightly. It also indicates that the concrete quality of the different TSTMs can be guaranteed to be similar to the greatest extent by being vibrated on the vibrating stand synchronously. During the design cooling and rapid cooling phases, temperature results of the four TSTMs and the environment show small differences. The maximal temperature difference between the surface and the internal concrete can be smaller than 1 ˚C when the cooling rate is 1 °C/h. Although four TSTMs are placed in the walk-in environment simulation laboratory, covering an area of approximately 40 square meters, the temperature can be controlled effectively as a pre-set. The temperature gradient of the four TSTMs is small, at 1 °C. Multiple temperature conditions, such as the semi-adiabatic temperature rise experiment for concrete, the design temperature history regulation, and rapid cooling can be implemented, which is particularly applicable for research and engineering.

- Temperature Stress Test Results of Multi-TSTMs

**Figure 14:**
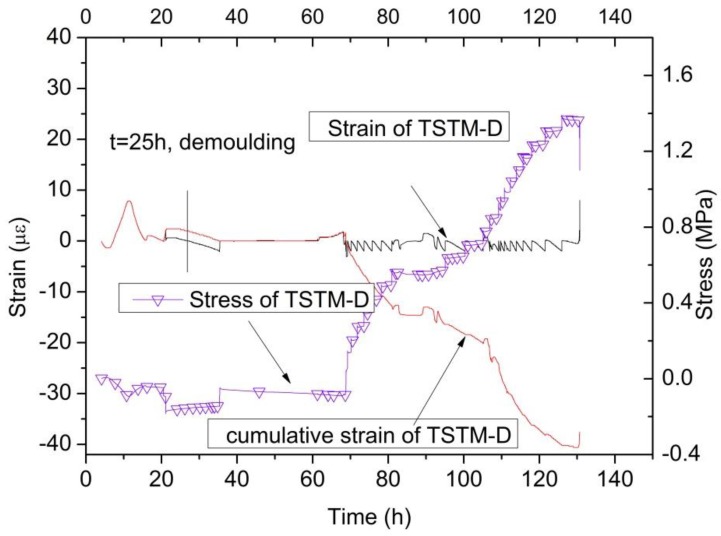
The stress and strain results of TSTM-D.

Four TSTMs are operated synchronously with different setting conditions. The specimen of TSTM-D cracks first when the temperature is reduced to 25.67 °C. The stress and strain curves are drawn in Figure 14. If the concrete is compressive, then the stress is expressed as a negative value. The strain is expressed as a negative value when the concrete shrinks, and vice versa. During the temperature following phase, the concrete swells as the concrete temperature increases. However, the stress increases only 0.1 MPa due to the effect of creep and relaxation. During the cooling phase, the concrete shrinks obviously. When the strain of the concrete exceeds 2 με, the motor adjusts the concrete to its original position and maintains the deformation at zero to achieve a 100% restraint degree condition. The failure stress of TSTM-D is 1.35 MPa and the cumulative elastic strain is 37.6 με, while the strain of free specimen on TSTM-A is approximately 230 με. The failure strain is small because the shrinkage strain transforms to creep strain.

**Figure 15:**
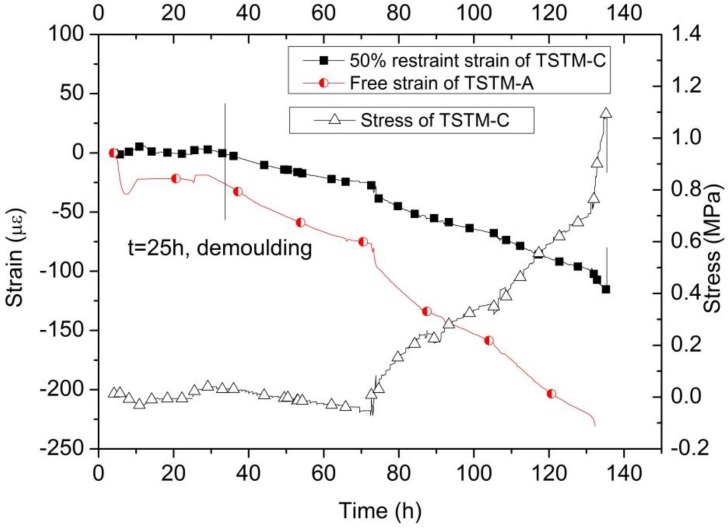
The stress and strain results of TSTM-A and TSTM-C.

The restraint degree of TSTM-C is 50% and TSTM-A is the reference free specimen. The cracking temperature of TSTM-C is 20.7 °C, which is 4.97 ˚C less than that of TSTM-D. The results show that the concrete cracks more easily under the strong restraint condition. The failure stress is 1.02 MPa and the failure strain is 115.75 με. The failure strain of TSTM-C is far greater than that of TSTM-D due to the different restraint degree. The creep strain is smaller than that of TSTM-D. The specific stress and strain curves are drawn in Figure 15, and the cracking results are listed in Table 4. Figure 15 shows that the strain of TSTM-C is almost 50% of TSTM-A at any time, which indicates that the self-developed TSTM system can simulate different restraint degrees and the reference specimen can be selected with other parallel specimens.

- E Results

**Figure 16:**
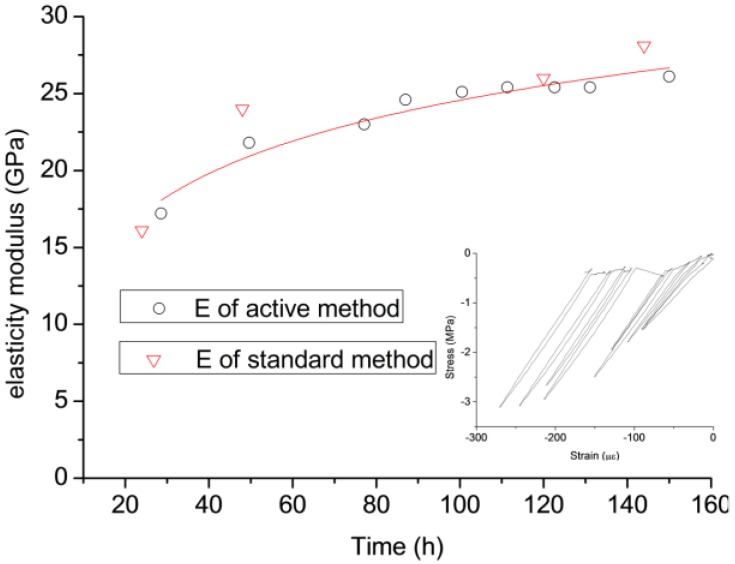
The compressive stress-strain curve for determining the elasticity modulus of TSTM-B.

TSTM-B is set as an almost free specimen to evaluate the elasticity modulus every 12 h. The E–time relationship is shown in Figure 16 and a fitting equation is listed as Equation (6):

(6) E=5.2×ln(t)+0.6165 (20 h≤t≤160 h; r=0.94285)

**Figure 17:**
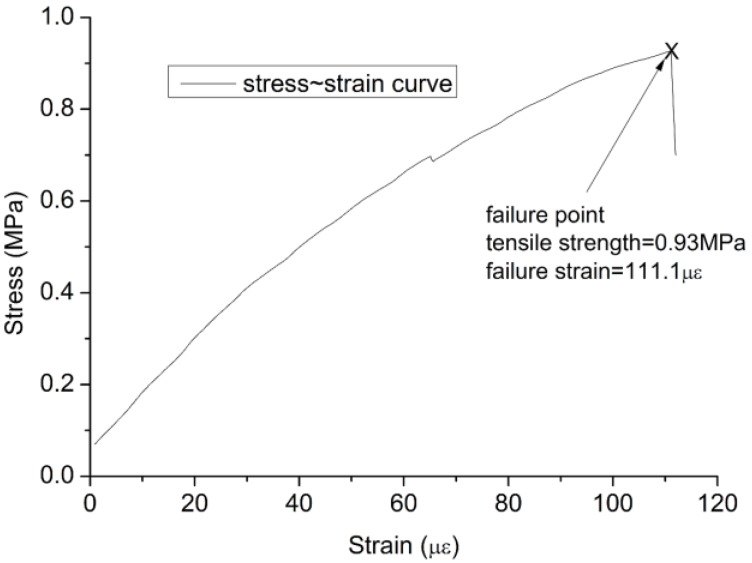
The tensile stress-strain curve of a specimen in TSTM-B.

The specimen is pulled to failure at the rate of 0.3 mm/min after the specimen of TSTM-C cracks; meanwhile, the concrete age is approximately six days. The stress-strain curve is drawn in Figure 17, in which the failure stress is 0.93 MPa and the failure strain is 111.1 με. The tensile elastic modulus derived from Figure 17 is approximately 8.8 GPa, but the compression elastic modulus of concrete is 26.1 GPa, determined by the active method on TSTM-B, shown in Figure 16. The difference between the results in tension and in compression may be because the sample is damaged during the active push process every 12 h. This can be supported by the phenomenon that the failure stress is only 0.93 MPa and only 68.8% of the failure stress of TSTM-D (1.35 MPa). However, the stress ratio is 68.8% of the free specimen/restraint specimen, which conflicts with the universally accepted research conclusion that the failure stress of the restraint sample is smaller than the free sample in the same experiment condition. The ratio of the stress to the tensile strength at failure is 0.6–0.9 for the restraint specimen [17,30,31,32].

The results indicate that the “active method of determining elastic modulus” may damage the sample. The micro-damage is not obvious in compression, but could be greatly affected in tension. The difference of E values derived from the active method and the standard method is small, so the active method is also an effective method. However, the active method is not recommended to be applied on the restraint specimen of the TSTM because the frequent tension and compression will influence the thermal and mechanical properties of the concrete under the effects of restraint and temperature. The E values of the active method and the standard method show little difference, indicating that the E value does not depend on the dimension of the sample [33], but could depend on the curing temperature of the concrete [34]. Thus, a specific TSTM, without any other application, such as a restraint test, could be used for determining E with the active method. The standard method is also practical for temperature stress testing under the same temperature and humidity conditions.

## 5. Conclusions

The self-developed synchronous closed loop federated control TSTM system is an effective device for researching the mechanical and thermal properties, and it has the following characteristics:

1. It has the comprehensive ability of simulating different restraint degrees, multiple temperature and humidity modes, and closed-loop control of multi-TSTMs during one test period.
2. The environment simulation laboratory system, including a walk-in environment simulation laboratory, allow for multi-concrete specimens of temperature stress tests to be conducted synchronously under the same environmental conditions. Additionally, no obvious fluctuation of the concrete temperature appears before or after concrete specimen demolding. The operation is more flexible during the test as the TSTM is no more restrained by the small chamber and fluid-cooled formwork. Finally, all parts of the TSTM are in the same environment, which reduces the disturbance caused by the external temperature.
3. The deformation measuring design with embedded cross part can obtain more accurate deformation and restraint degree results with little damage.
4. The compensation method of temperature deformation with different considerations of steel materials can eliminate the external temperature deformation’s effects on the concrete specimen.
5. Some design concepts, such as the sliding sleeve on the steel shaft, height-adjustable ball supporting points, cross-head positioning fixture, and the mold assembly platform, have been taken into consideration so that the self-developed TSTM has greatly overcome the difficulty of eccentric force and deformation.
6. The detachable mold design of TSTM guarantees that the concrete quality of different TSTMs is similar to the greatest extent by being vibrated on the vibrating stand synchronously.
7. The active method should be used for determining E on a specific TSTM without any other application, such as restraint testing, because the frequent tension and compression will cause damage which will influence the mechanical properties in tension.

## Figures and Tables

**Table 1:** The performance parameters of the self-developed TSTM system.

TSTM Performance	Capacity
Restraint degree	0%–100%
Loading capacity	±200 kN
Temperature control capacity	−20 to +80 °C
Humidity control capacity	0–100% RH
The diameter of the steel shaft	130 mm
Specimen dimensions	150 mm × 150 mm × 2000 mm

**Table 2:** The mixing ratio of concrete specimens for the TSTM experiment (kg/m^3^)

Water	Cement	Sand	Gravel
200	380	900	975

**Table 3:** The constituents of the Portland cement.

Compound	CaO	SiO_2_	Al_2_O_3_	MgO	SO_3_	Fe_2_O_3_	Na_2_O	K_2_O	TiO_2_	P_2_O_5_
Mass Percent/%	47.87	25.12	11.29	5.52	2.95	2.39	0.654	0.599	0.399	0.247

**Table 4:** Cracking results of TSTM experiments.

TSTM	B	C	D
**Tensile Strength (MPa)**	0.93	1.02	1.35
**Failure Strain (με)**	111.1	115.75	37.6
**Cracking Temperature (°C)**	-	20.7	25.67

## Supplementary Materials

The following are available online at http://www.mdpi.com/1996-1944/10/4/419/s1, Video S1: Mold assembling process, S2: Hoisting process, S3: Demolding process.
